# Radiation recall pneumonitis from immune checkpoint inhibitors after extra pulmonary radiation

**DOI:** 10.1002/rcr2.1223

**Published:** 2023-09-19

**Authors:** Leslie E. Kao, Diane Stover

**Affiliations:** ^1^ Pulmonary Medicine Service Memorial Sloan Kettering Cancer Center New York New York USA

**Keywords:** anti‐PD‐1/PD‐L1, cancer, pneumonitis, radiation recall pneumonitis

## Abstract

Radiation recall is an unpredictable, poorly understood inflammatory reaction within the confines of previously irradiated tissue that occurs following exposure to a systemic agent. In this article, we will focus on a subcategory of radiation recall, called radiation recall pneumonitis (RRP), precipitated by immune checkpoint inhibitors (ICI). Historically, RRP can develop weeks to years after radiation treatment to the lung, most commonly after receiving certain chemotherapeutic agents, but more recently has been recognized in association with immunotherapy agents (ICIs). Up until now, RRP following exposure to ICIs has been described in patients who have received radiation to the lung itself. Here we present three cases of RRP in patients who received radiation to areas adjacent to the lung parenchyma, and developed pulmonary infiltrates in an area adjacent to the radiation field while on ICI therapy. Since ICI induced RRP is treatable and steroid sensitive, early recognition and treatment are important.

## Graphical abstract

Radiation recall is an unpredictable, poorly understood inflammatory reaction within the confines of previously irradiated tissue that occurs following exposure to a systemic agent. In this article, we will focus on a subcategory of radiation recall, called radiation recall pneumonitis (RRP) precipitated by immune checkpoint inhibitors (ICI). Up until now, RRP following exposure to ICIs has been described in patients who have received radiation to the lung itself. Here we present three cases of RRP in patients who received radiation to areas adjacent to the lung parenchyma, and developed pulmonary infiltrates in an area adjacent to the radiation field while on ICI therapy. Since ICI induced RRP is treatable and steroid sensitive, early recognition and treatment are important.

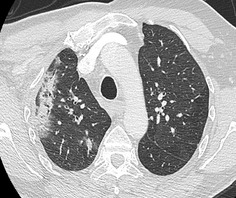

## INTRODUCTION

Radiation recall is an unpredictable, poorly understood inflammatory reaction within the confines of previously irradiated tissue that occurs following exposure to a systemic agent anywhere from weeks to months and even years after irradiation. In this article, we will focus on radiation recall pneumonitis (RRP) precipitated by immune checkpoint inhibitors (ICI).

Radiation recall injury was first described in 1959 by D'Angio et al.^1^ with a case of reactivation dermatitis following actinomycin D therapy. Subsequently, most cases of radiation recall described in the literature have been cutaneous in nature, and most commonly involve chemotherapies such as doxorubicin, gemcitabine, capecitabine, adriamycin and taxanes, although antibiotics such as quinolones have also been reported. More recently, molecularly targeted therapies such as sorafenib, tamoxifen, erlotinib and osimertinib have been implicated.^2^, ^3^

RRP is a subcategory of radiation recall that involves previously irradiated lung fields that has been historically attributed to chemotherapies as mentioned above.^2^, ^3^ RRP can cause severe pulmonary symptoms, leading not only to treatment discontinuation but also long‐term steroid therapy. Aside from chemotherapy, antibiotics and small‐module tyrosine kinase inhibitors, ICIs have more recently been associated with RRP.^4^

ICIs, first FDA approved in 2011, have become standard treatment for a myriad of locally advanced and metastatic cancers by harnessing the intrinsic immune response of T‐cells against tumour antigens with the sometimes‐unintended consequence of T‐cell attack on self‐antigens. This can cause a spectrum of adverse events independent of other (neo‐) adjuvant therapies, including immune checkpoint inhibitor pneumonitis, the most frequently described ICI adverse event in the lung.^5^, ^6^ However in 2017, Shibaki et al. published two patient case reports of RRP associated with nivolumab, an anti‐programmed cell death‐1 (anti‐PD‐1) antibody ICI.^7^

Since the original reports in 2017, several case series have been published with regards to ICI‐induced RRP involving not only nivolumab, but other anti‐PD‐1 therapies, such as pembrolizumab.^3^, ^8^, ^9^, ^10^, ^11^ Although the current incidence is unknown, two retrospective observational studies have been published. Cousin et al. reported 15 patients with RRP out of 112 patients from a single institution who received both radiotherapy (RT) to the lung and ICI drugs.^12^ Pozzessere et al., in a retrospective analysis, found 6 patients out of 253, who received RT followed by ICI, and developed pneumonitis within the previously radiated field.^13^ The reported intervals for the development of RRP following ICI therapy can be from weeks^3^ to years^7^ after the RT. Up until now, RRP following exposure to ICIs has only been described in patients who have received radiation to the lung itself. Here we present three cases of RRP in patients whose RT was outside the lung and who developed pulmonary infiltrates in an area adjacent to the radiation field while on ICI therapy.

## CASE SERIES

### Case 1

**FIGURE 1:**
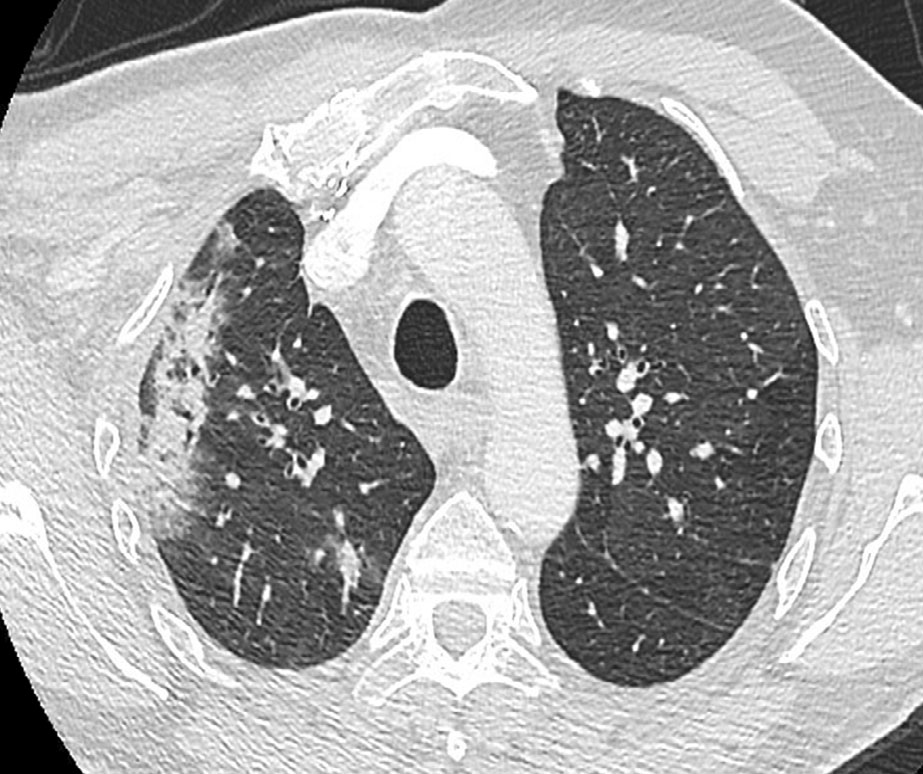
Computer tomography chest without contrast with right upper lobe consolidation.

A 71‐year‐old man, a former 15‐pack‐year smoker, was diagnosed with local cutaneous squamous cell carcinoma of the mid‐upper anterior chest in April 2018. He received 30 fractions of radiation to the right axilla, right infra‐clavicular and supra‐clavicular regions in June to July 2018 for a total of 60 Gy. Metastatic disease to the bilateral lungs and right adrenal gland was found in November 2018 and the patient was subsequently started on systemic cemiplimab in December 2018. In January 2018 after his third cycle, the patient began to develop worsening dyspnea with exertion and mild rash on his palms and feet. Chest computer tomography (CT) scan showed new opacity with air bronchograms in the right upper lobe (RUL) in the area adjacent to previous right axillary radiation (Figure 1). All subsequent doses of cemiplimab were held due to concerns for grade 1 pneumonitis. Despite discontinuation, the patient's symptoms persisted, and 3 months after symptom onset, he was started on prednisone 100 mg (1 mg/kg dosing) daily and slowly tapered off over 9 months with no recrudescence of his pneumonitis. Radiographic imaging showed progressive improvement in the consolidation, starting 1 month after steroid initiation and corresponding with symptomatic improvement. The patient remains well 4 years after discontinuing the steroids and has not received any further ICI therapy.

### Case 2

**FIGURE 2:**
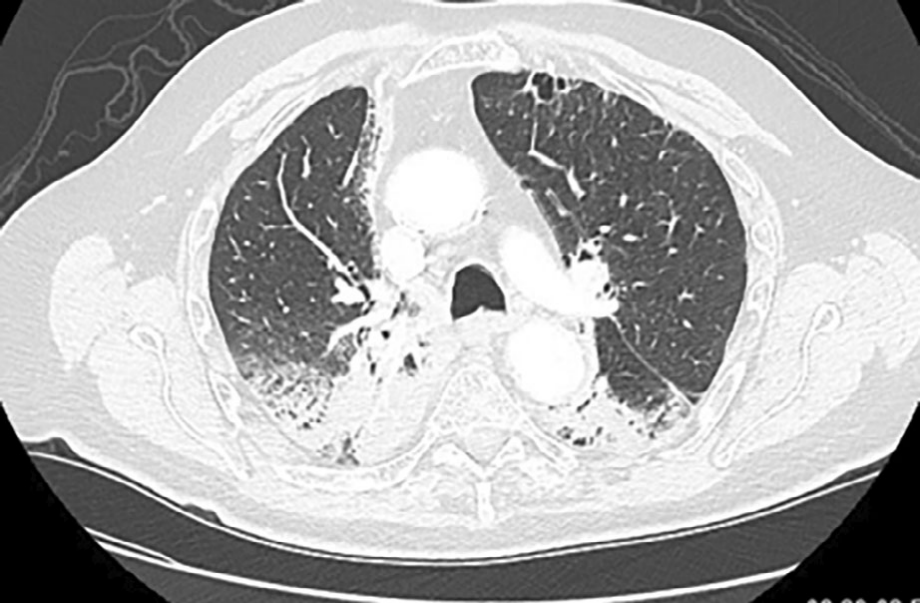
Computer tomography angiography chest showing bilateral posterior upper/lower lobe consolidation at vertebral level T4.

A 75‐year‐old man was diagnosed with bulky metastatic Merkel cell carcinoma to his right superficial groin in March 2017. Seven months after diagnosis, the patient received RT to his thoracic (T2‐T8) vertebrae for moderate cord compression from paraspinal metastasis. Two weeks later, he was started on systemic avelumab (10 mg/kg infusion every 2 weeks). After his tenth avelumab infusion and 5 months after his last radiation therapy, the patient developed progressive shortness of breath. Evaluation did not reveal any infectious aetiology or worsening metastasis. CT chest and PET scan were significant for bilateral, dense consolidations in the regions adjacent to the previous RT (Figure 2). These consolidations were new compared with the last chest CT scan which was done after the RT to the paraspinal area. After an empiric course of antibiotics without improvement, he was started on 60 mg of prednisone with a slow taper over 6 months. Symptoms resolved back to the prior baseline with the corresponding radiologic resolution, including a decrease in FDG avidity of paraspinal consolidations. The patient remains on surveillance imaging 4 years after diagnosis of pneumonitis without recrudescence. He has not received any further ICIs.

### Case 3

**FIGURE 3:**
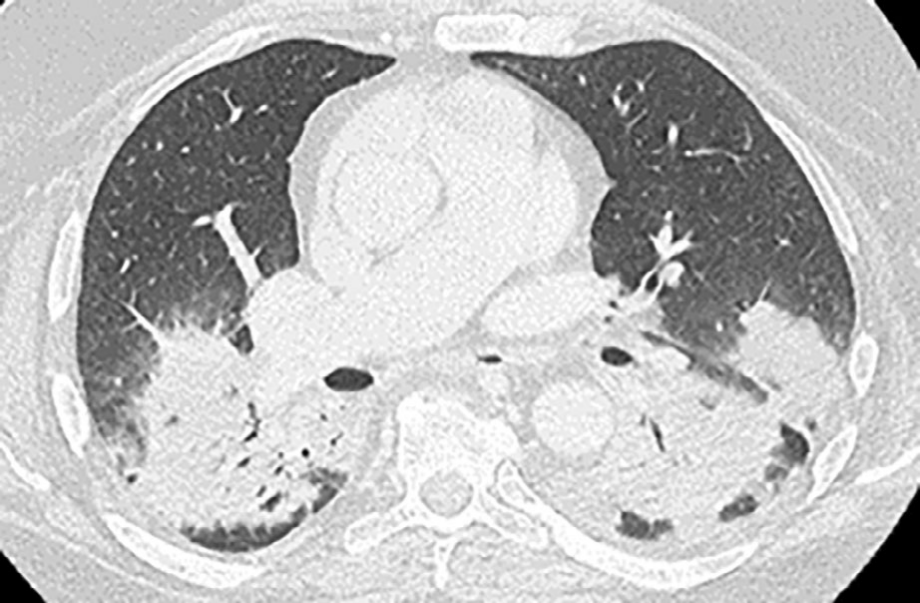
Computer tomography angiography chest with bilateral lower lobe para‐vertebral consolidations at vertebral level T6.

The final patient is a 68‐year‐old man with renal clear cell carcinoma and epidural disease, treated with radiation to T6 and T9‐11 vertebrae, followed 3 months later by adjuvant nivolumab and ipilimumab. Three months after initial radiation therapy and after his fourth ICI cycle in September 2022, the patient developed subacute dyspnea. CT chest showed new bilateral paravertebral findings in the right upper lobe and left lower lobe adjacent to the patient's radiation field (Figure 3). These infiltrates were not present radiographically after the spinal radiation and appeared after the ICI therapy. Peripheral infectious work‐up was negative. Pulmonary function testing showed a severe restrictive deficit. A 6‐week taper of daily prednisone (0.5 mg/kg) was started with a resolution in symptoms and significantly decreased consolidations in radiographs. More than 6 months later, since completion of the steroid taper, the patient remains symptom‐free with no recrudescence clinically or radiographically.

## DISCUSSION

Here we report three patients who received radiation treatment to extrapulmonary sites and developed pneumonitis following ICI treatment. Imaging at symptom onset showed new consolidation restricted to the area of the lung adjacent to the area of the radiation.

We hypothesize that the lung received radiation as an ‘innocent bystander’ and RRP was unmasked by the ICI treatment in a two‐step or ‘two‐hit’ process. At the time of the initial radiation, the lung parenchyma may have received a low‐dose of radiation which did not reach an inflammatory threshold for clinical detection. This caused a pre‐existing vulnerability, which was then potentiated and activated by the immune augmentation created by the ICI drugs leading to clinically detectable pneumonitis. Differing from the diffuse presentation of immune checkpoint pneumonitis, this ICI associated RRP was only radiographically detectable in a well‐demarcated anatomical region corresponding to the previous field of radiation—the only area of lung parenchyma that received both inflammatory hits. The well‐demarcated areas on the CT scans, especially in Case 1, demonstrate the geographical nature of RRP with the infiltrates localized to the area of the radiation rather than a diffuse, multi‐lobar pattern in ICI pneumonitis.

In addition, the presentation in these cases seems distinct from radiation pneumonitis alone based on the temporal association with ICI treatment, and the fact that the lung was not radiated directly. While RT pneumonitis is sometimes seen without direct pulmonary radiation, most frequently when the spinal areas are radiated for paraspinal disease, our patients did not have clinically relevant symptoms or radiographic findings until after ICI initiation, making RRP more likely. Finally, while none of these cases were biopsy‐proven, their clinical symptoms, radiographic changes and subsequent improvement after systemic steroid treatment suggest an inflammatory pneumonitis rather than an infectious or malignant aetiology.

The presentation of RRP with ICI therapy up until now has only been described in patients who received radiation directly to the lung parenchyma. The three patients that we describe here never received direct radiation to the lung—only radiation to areas adjacent to the lung parenchyma. Since ICI induced RRP is treatable and steroid sensitive, early and timely recognition and treatment are important. We give evidence here that extrapulmonary radiation in locations close to the lung but not directly to the lung parenchyma followed by ICI can cause RRP.

## AUTHOR CONTRIBUTIONS

All authors discussed the results and contributed to the final manuscript.

## FUNDING INFORMATION

This research was funded in part through the NIH/NCI Cancer Center Support Grant P30 CA008748.

## CONFLICT OF INTEREST STATEMENT

None declared.

## ETHICS STATEMENT

The authors declare that appropriate written informed consent was obtained for the publication of this manuscript and accompanying images.

## Data Availability

Data available on request due to privacy/ethical restrictions.
